# The “First daughter” effect: Human rights advocacy and attitudes toward gender equality in Taliban-controlled Afghanistan

**DOI:** 10.1371/journal.pone.0298812

**Published:** 2024-07-17

**Authors:** Kristina Becvar, Charli Carpenter, Bernhard Leidner, Kevin L. Young

**Affiliations:** 1 Data Analytics and Computational Social Science (DACSS) Program, College of Social and Behavioral Sciences, University of Massachusetts Amherst, Amherst, MA, United States of America; 2 Department of Political Science and Legal Studies, University of Massachusetts Amherst, Amherst, MA, United States of America; 3 Department of Psychological and Brain Sciences, University of Massachusetts Amherst, Amherst, MA, United States of America; 4 Department of Economics, University of Massachusetts Amherst, Amherst, MA, United States of America; Tokyo Medical and Dental University: Tokyo Ika Shika Daigaku, JAPAN

## Abstract

International concern for the human rights of Afghan women has spiked since the Taliban consolidated power in Afghanistan in fall 2021. Yet little is known about how to effectively advocate for women’s human rights under this new context. We present findings from a random sample of all adult Afghan internet users’ attitudes toward peace, security, gender, and human rights and find significant support for women’s human rights as a national priority within Afghanistan, even when controlling for other priorities and even among many men and women aligned with the Taliban. Given that men now have much more political power in Afghan society to protect women’s rights, we paid particular attention to men’s attitudes toward women’s human rights. Our evidence from an embedded survey experiment, building on earlier literature from other countries, demonstrates that fathers of eldest daughters are particularly likely to favor prioritizing women’s rights when primed to think about the gender of their eldest children. Thus, the human rights and humanitarian community should spend more time and attention engaging with this demographic, and specifically creating marketing and advocacy strategies that encourage men to think about or act on behalf of their eldest daughters.

## Introduction

International concern for the human rights of Afghan women has spiked since the Taliban consolidated power in Afghanistan in the fall of 2021 [1]. Following that event, civilians (particularly women and LGBTQ persons, children, activists, ethnic minorities, former interpreters, and development/ aid workers) have been targeted with violence, repression and imprisonment [2–4]. In their first few months of control, there was significant evidence of the Taliban violating the rights of women and girls across a number of categories, including education, employment, freedom of movement, dress, gender-based violence, access to healthcare, and sport [5, 6]. Over the past years, the Taliban have engaged in house raids aimed in part at terrorizing women and dissidents, brought back draconian punishments, excluded girls from schooling beyond sixth grade [7], and excluded women from employment in the NGO sector [8].

International actors engaged in interventions in the country are attempting to balance their efforts between those demographic groups a) most motivated to support women’s rights and b) most possessing some power in Afghan society with which to do so. This poses important information, strategic, and normative dilemmas for the international community [9, 10]. On the one hand, some political science research shows that a combination of ideational pressure and economic incentives can create improvements in governments’ human rights performance, including gender equality [11]. On the other hand, some literature suggests that coercive international pressure for women’s human rights in non-Western contexts can generate a backlash against women and human rights, and fuel conflict dynamics [12]. In this context, aid agencies often desire to identify and support initiatives and activism that are local and culturally authentic. But little is known about what sort of international support Afghans most want, or which demographics are likeliest to be the key advocates of gender equality initiatives.

Given that men rather than women now have much more political power in society to protect women’s rights, we sought to investigate the determinants of male attitudes on gender equality. In particular, two embedded survey experiments enabled us to test a hypothesis drawn from US-based studies of men’s attitudes toward gender equality, but which has not yet been tested in contexts characterized by extreme gender inequality or gender apartheid: the hypothesis that fathers of eldest daughters are particularly likely to favor prioritizing women’s rights. The ‘first daughters’ effect. We find positive evidence for the first daughters effect, which is particularly striking given the nature of Afghan gender politics.

Our findings add to existing research on first-daughter effects, and in a particularly striking context of radical gender inequality. Our analysis also deepens the analysis of first daughter effects by differentiating what mechanisms might be driving it. Using a priming experiment, we differentiate the source of the first-daughter effect as emanating from two different mechanisms. An ’experiential’ mechanism indicates that men *simply having* a daughter as their first child—i.e., their direct experience within having a first daughter–can potentially drive the first daughter effect. A ‘priming’ mechanism indicates that the direct experience of having a first daughter is not sufficient but has to be *activated in their mental schema* for the first-daughter effect to occur. In other words, when having an eldest daughter is *actively recalled* through question wording order, it drives the first-daughter effect. It is thus not the ‘experiential’ effect of merely having an eldest daughter alone which shapes attitudes, but the active recall or ‘priming’ of men’s status as a father of an eldest daughter.

Empirically, we differentiate these mechanisms through a ‘priming’ manipulation, whereby half of all respondents receive questions about the gender of their eldest child before the attitudinal outcome question regarding women’s human rights, and half after. This simple feature of our survey instrument allows us to pinpoint the relative contribution of the ‘experiential effect’ versus the ‘priming’ effect. We find the priming effect to be the key mechanism: fathers of eldest daughters are especially likely to argue in favor of gender equality if they are first encouraged to think about the gender of their eldest children. (By contrast, fathers of eldest sons are neither more nor less likely to support gender equality if asked to think about their eldest children before answering the same question.) This has significant implications not only for scientific research on gendered attitudes in society but, as we discuss below, also for interventions designed to improve the lives of women and girls in Afghanistan and potentially other states with radically unequal gender orders.

In what follows, we first briefly discuss some earlier scientific findings on which our project is based, specifically the literature on the impact of gender and children’s birth order on parents’ attitudes toward gender equality in society. We then discuss the methodology by which we collected and analyzed data on Taliban-controlled Afghanistan and present our preliminary findings specifically on the relationship between the gender of an eldest child and fathers’ likelihood to support gender equality. In short, we find that priming men to think about their eldest daughters has an important impact on their attitudes toward women’s rights as a priority for the country’s future.

### Background and literature review

An emerging literature on the determinants of male attitudes toward gender equality suggests that the gender of one’s eldest child can influence men’s attitudes and behaviors toward women and gender. In particular, emerging literature in the US, UK, Australia, and Canada suggests that fathers of eldest daughters are a demographic particularly attuned to and supportive of women’s human rights both behaviorally [12–15] and attitudinally [16–20]. However, we know little about whether these findings travel to non-Western contexts or how to explain them.

Behaviorally, some research shows that fathering daughters affects men’s political behavior in ways conducive to gender equality. Research on U.S. parents in 1999 showed that both fathers and mothers showed increased support for policies designed to address gender equity when they had daughters only, with the effect stronger for fathers than mothers [12]. Building on that research, specific analysis of U.S. lawmakers revealed that having a daughter increases a male congressperson’s propensity to vote liberally on issues such as reproductive rights issues [13]. The U.K. research analyzed voting patterns specifically, finding that first time fathers of daughters move toward left-wing political parties, and further found that having a son led to favoring right-wing parties. The results were corroborated by microdata from a German panel [14]. A 2019 study of household surveys from several countries found that men who have a firstborn daughter instead of a firstborn son are 3.4 percent less likely to be violent with their partner each year [15].

The attitudinal research on fathers of daughters stems from studies on Canadian and U.S. parents where both fathers in Canada and mothers in the U.S. and Canada had increased support for policies designed to address gender equity when they had daughters only [18]. Longitudinal studies from the U.S. [19] and the U.K. [17] build on the first daughters research and introduce birth order and age to the research. A U.S. study found that first time parents support more progressive gender roles among fathers, but not mothers [19]. The U.K. study found that the effect was driven by fathers of school-aged daughters, consistent with exposure and identity theories [19].

There is no consensus on the cause of these phenomena. Research has controlled for the age when men became fathers as well as whether the first child’s birth was before Title IX in the U.S., finding no change in the effects [16]. It is believed that preparation for parenthood is when men undergo a process of self-socialization. These changes can incur cognitive dissonance and subsequent changes to beliefs and behaviors [20]. However, because the various studies do not use consistent independent variables it is difficult to establish conclusively what is driving these different results or how robust or universal they are. And importantly, none of these studies explore the possibility that asking men to acknowledge the gender and/or birth order of their children could be affecting the findings themselves.

Moreover, with few exceptions [21, 22] the first daughters hypothesis has not yet been replicated widely outside Western cultural contexts. Research on fathers of first-born daughters in Turkey showed a significant effect on marital domestic violence against women but did not analyze attitudes [23]. Research on South African fathers did focus on attitudes but found no significant effect of having a daughter overall or having a daughter first on a father’s gender views [22]. A study in Japan also found the results did not hold the same significance as in the US, highlighting the importance of considering how structural characteristics within a society affect gender attitudes [24].

Our project is the first to investigate the first-daughter effect in Afghanistan both to see whether the attitudinal finding can travel to non-Western societies and as a potential clue to where levers in society may tip in favor of improvements in women’s human rights. This is an especially significant institutional context to investigate the first-daughters effect not only because of the salience of gender issues in Afghanistan but also because it has experienced a recent dramatic shift given the takeover of the Taliban following the American withdrawal. We simply do not know whether such a finding would apply in contexts characterized by extreme gender inequality such as Taliban-controlled Afghanistan.

However, if such a finding did hold–and particularly if it could be activated through priming men to think about their daughters–it would be of immense value to advocates of women’s human rights and international service-delivery organizations, who face a dilemma in Taliban-controlled Afghanistan and similar contexts [25]. On the one hand, data shows a positive correlation between gender equality, development, security, and peace both within and among nation-states [26], and international advocacy and incentives can play an important role in supporting and creating space for local human rights advocacy groups [11, 27–29]. On the other hand, outside advocacy for women’s human rights can cause a backlash [30], feed ongoing conflict dynamics [31, 32], result in further restrictions on women’s civil society organizations [33, 34] or at best create marginal gains for women [35]. For example, one study of male attitudes toward gender equality in Afghanistan revealed a tendency for Afghan men to feel defensive about non-Afghans dictating gender norms, activating defensive hyper-masculinity [36].

Recognizing this kind of dilemma, human rights and development organizations generally recognize that the most effective international programming builds on the strengths of local communities and existing community resources to be effective and sustainable [37]. Still, international actors often misunderstand the complex balance conflict-affected civilians in specific cultural contexts like Afghanistan must navigate [38, 39]. As a result, there is often a disconnect between international and local agendas, leaving international actors wondering how to help without harming.

In Taliban-controlled Afghanistan, these well-documented tendencies have historically been exacerbated by two problems. First, there has been a severe shortage of data, especially since the withdrawal, on which Afghans want what and which demographics would be most receptive to working with international human rights groups to promote women’s rights. The most rigorous public opinion polling institution in the country, Asia Foundation, evacuated their personnel during the withdrawal due to security concerns, and the most recent report from the Kabul-based Organization for Social and Research Analysis pre-dates August 2021 [40, 41]; a Gallup poll in July/August 2022 focused primarily on attitudes toward the US withdrawal [42].

This reflects a wider problem in the international development and advocacy community: many “Afghan voices” amplified by international donors and stakeholders are either members of the diaspora or English-speaking elites embedded in civil society organizations funded by the donors themselves [43]. Snowball samples that begin with such starting points tend to be biased in favor of Western views [44]. Efforts to collect Afghan public opinion from social media scraping are biased in favor of those who would willingly share their views on social media, which unsurprisingly skew toward those who support the Taliban [45]. The scarcity of cross-sectoral national-level data on wider Afghan attitudes toward gender equality and the future of their country, disaggregated by gender and other important controls such as education, political alignment, religiosity, geographical region, and socio-economic status, has stymied effective global policymaking and advocacy, particularly since the withdrawal. Nonetheless, understanding the views and needs of these civilians in all their complexity is critical in making recommendations to those involved in constructing both international humanitarian response and policies that can help stabilize the country.

We examined not only the beliefs of a wide swath of Afghans disaggregated by gender and other important demographic variables, but also whether certain variables known to affect men’s attitudes toward gender specifically might be at work in Afghanistan in ways that would provide especially effective inroads for international gender equality advocates. Our finding–that the gender of the eldest child itself has only a limited effect on attitudes but that *priming respondents on this question has a substantial effect*–both yields practical implications for women’s rights advocates and advances the literature on men’s attitudes toward gender equality.

## Materials and methods

To understand current Afghan attitudes and identify those Afghans most receptive to Western engagement efforts, we surveyed a random sample of Afghan internet users on a variety of peace, security, gender, and human rights issues. Our hypothesis was that attitudes toward prioritizing peace, security, gender, and human rights in Afghanistan would vary by gender, age, ethnic group, geographic location, and (given the findings of earlier literature on fathers) the gender of the eldest child.

We hired a global survey firm, RIWI, to conduct a cross-sectional survey of adult Afghan internet users, collecting data in three waves from March 16 through June 16, 2022. As only ~23% of Afghans are internet users and ~68% of Afghans had cellular mobile connections circa January 2022 [46], this is not a representative sample of the entire population of Afghanistan. However, the security situation in Afghanistan informs the choice of the RIWI methodology as the most secure population from which to obtain this important data. Moreover, the method is advantageous in that it allows a completely random sample of this population.

RIWI’s patented Random Domain Intercept Technology (RDIT) allows the collection of a random sample of all internet users in a geographic region. It works by intercepting “broken links” that normally take users to an error message, but instead, they are invited to opt-in to a survey on a site temporarily controlled by RIWI [47]. Because any internet user has an equal chance of surfing to such a link, this technology corrects for other sampling biases typical in conflict zones or internet research and allows us to access hard to reach and vulnerable populations. For example, the survey interface does not include ad-tracking pixels, so any ad-blocking technology does not materially reduce the size or diversity of the sample [48]. This particular method had been used by a range of other studies to acquire hard to reach populations in a systematic way [49–51].

Participants’ security is paramount to collect candid answers in conflict zones and totalitarian contexts. We received clearance from University of Massachusetts’ Institutional Review Board on December 13, 2021 (Protocol # 3132). Upon discovering the survey, participants chose whether to safely and anonymously opt-in to take part in the study, informed they could end their participation at any time and that no personally identifiable information was stored or reported, including IP addresses. Due to the ability of the participants to exit the survey at any time, the phenomenon of incentive bias was eliminated [49].

The RIWI interface offered the survey in relevant language choices. Of the Web-users who landed on the RIWI-operated inactive Web domain, 26,039 opted to begin the survey, with over 3,020 completing all questions and 7,513 completing the question on the dependent variable, support for women’s human rights. The respondents’ gender represented significantly more males (n = 18,216) than females (n = 8,116). We know that of internet users in the country, urbanites, men, and younger people are over-represented, so answers were weighted against the most recent census. Respondent weight values are generated post-stratification using a raking algorithm and are applied to the entire dataset of respondents who opted-in to the survey. Raking, otherwise known as iterative proportional fitting, chooses a set of variables where the population distribution is known, and iteratively adjusts the weight for each case until the sample distribution aligns with the population for those variables. For example, the process will adjust the weights so that e.g. a gender ratio for the weighted survey sample matches the desired population distribution, and do the same for geographic region, etc. Raking is the standard weighting method used by many other public pollsters [52]. We include results with and without survey weights in our analysis and in the S1 Appendix.

All participants received questions on physical safety, security, food security, support for and legitimacy of the Taliban, opinions on frozen central bank reserves, the role of the international community in supporting Afghans, personal priorities, and women’s human rights. We also gathered data on a range of demographic variables including education, socio-economic scale, urban v. rural residence, age, gender, language, religiosity, and marital status.

In addition, among the demographic questions asked in the survey, Question 1 asked, “If you have children, what is the gender of your oldest child?”, with the responses representing whether it was a daughter, a son, or if the respondent had no children. For the purposes of the result we are reporting here, this becomes our key explanatory variable. We did not include information on the number of children or the specific ratio of children, as we sought to make this question–and thus the potential priming effect–as simple as possible. Question 9 asked, “How much would you agree or disagree with the following sentence: “I believe achieving human rights for women is among the top priorities for the future of my country”?” The response to this question was a 5-point Likert scale with choices, “strongly disagree”, “disagree”, “undecided”, “agree”, and “strongly agree.” To conduct our analysis, we converted these variables to indicate either agreement (either ‘agree’ or ‘strongly agree’) or otherwise. This question is the key dependent variable for this analysis.

We also augmented this basic research design in two ways. First, we implemented a two-group experimental design in which participants were randomly assigned one of two versions of the survey with the questions in a specific order to allow us to test not only whether having an eldest daughter influenced their attitudes but also whether being asked to think about the gender of their eldest child influenced their attitudes on other questions. Specifically, 50% received version one, which offered the question of the gender of the respondent’s eldest child at the beginning of the survey, and 50% received version two, which offered the question of the gender of the respondent’s eldest child at the end of the survey.

Open-ended questions were translated using Google Translate and analyzed by a team of student coders with DiscoverText data analysis software [53], using a combination of grounded theory and the application of auto-coded metadata. Inter-rater reliability was assessed after each round of coding using Fleiss’ Kappa. This yielded not only a set of annotated open-ended data on how Afghans view gender equality but also the ability to examine the influence of demographics or experimental treatments on respondents’ open-ended explanations of their answers, as well the answers themselves. The results of these analyses are described below.

## Results

We found significant support for women’s rights overall in Taliban-controlled Afghanistan. 66% of Afghans “agree” or “strongly agree” with the statement, “I believe achieving human rights for women is among the top priorities for the future of my country,” and the largest group “strongly agreed” (42%). This finding holds even when disaggregated according to support for the Taliban government: even among those who support the Taliban government “a lot,” 45% also “strongly agree” that achieving human rights for women is a priority for the future of the country.

Of the 66% who leaned toward agreeing with the statement on women’s human rights as a top priority, 2061 Afghans explained their answer in their words when asked, “What would achieving women’s human rights look like for you?” Many of them described the specific rights that were most important to them, including education, livelihoods, political participation and “other rights” such as the right to play sports. Others commented that respecting women’s rights would lead to a stronger society or emphasized an improved economy as important to women’s rights, and many spoke of the complementarity between Islam and women’s rights. Some articulated a vision of a country without Taliban rule, saying no women’s rights were possible under the Taliban, and others called explicitly for international pressure or intervention to restore women’s fundamental freedom.

We wanted to know what predicted individuals ending up in that 2/3 majority who “agreed” or “strongly agreed” with women’s human rights, versus those who didn’t–and in particular, whether being a man, a parent, and reporting having a daughter as an eldest child versus a son as an eldest child made a difference when controlling for other factors.

We first ran regressions to assess the correlates of supportive attitudes toward the human rights of women on various measures, using a logistic regression model in which the dependent variable is a simple indicator of whether a respondent did or did not agree/strongly agree with the statement “I believe achieving human rights for women is among the top priorities for the future of my country.” We also deployed an ordinal logistic regression model in which each value of the Likert scale is considered, from ‘strongly disagree’ to ‘disagree’ to ‘undecided’, ‘agree’ and ‘strongly agree’. Following a Brant [54] test, we found that the parallel trends assumption holds. We report here only the logistic regression results, which are easier to interpret.

Our first ‘baseline’ model uses all respondents in our sample and includes a range of covariates that could plausibly be associated with attitudes toward women’s rights. These include the gender of the respondent; the level of support for the government; the religiosity of each respondent and their families; the respondents’ residency in an urban or rural area; levels and different forms of education; and whether the respondent was single, widowed or divorced, where being married was the reference category. We included age-range dummy variables for these models, and main results are not sensitive to this specification or the under/over 30 dummy variable alternative. We also include a measure of socio-economic status in terms of perceived wealth and poverty of a respondent’s family relative to others, using the Macarthur Scale of Subjective Social Status [55]. A final additional variable we included was a respondent’s level of agreement with the notion of human rights as a general priority. We also controlled for potential time-effects by including a dummy for each ‘wave’ of the study.

**Fig 1:**
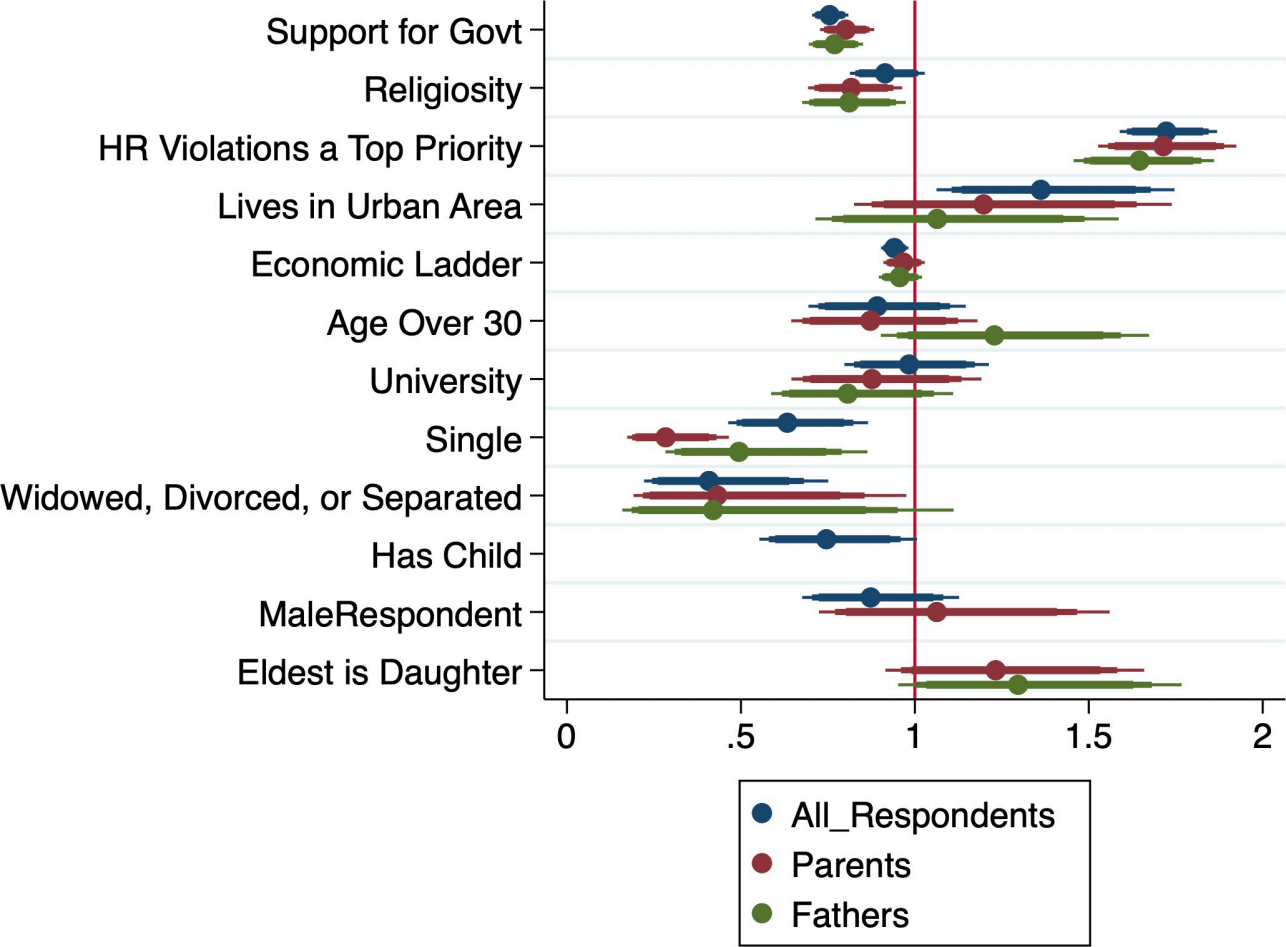
Coefficient plot estimates of support for gender equality in Taliban-controlled Afghanistan.

Fig 1 visualizes our first findings via a coefficients plot. This first model (in blue) can be interpreted as the baseline model for all respondents, without examining first daughter effects and against which those effects can be compared. We include the full regression table results in the S1 Appendix but visualize the results here in order to show visually the relative magnitude and significance of different kinds of variables.

We find in the ‘All Respondents’ model results that women respondents are more likely to support human rights for women than are male respondents. Those living in urban areas show more support, and those perceiving themselves as ‘higher’ on the economic ladder are also less likely to be supportive. Support for the Taliban government in power, and one’s family religiosity, are both negatively associated with support for the human rights of women. Other associated beliefs, such as human rights as a top priority for the government, are very strongly associated with support for women’s rights.

The ‘Parents’ model then includes the main variables of interest, namely whether or not the respondent has children and/or an eldest child who is a daughter. The question was worded to respondents as “If you have children, what is the gender of your oldest child?” The options were “My oldest child is a daughter”, “My oldest child is a son” and “I have no children”. We operationalize this as two separate dummy variables for daughters and sons, respectively, while having no children is the reference category.

A ‘Fathers’ model then restricts the same sample to only men with children since this is where the eldest-daughter effect is widely found to be manifest [14, 16, 19] (see, however, [17]). These results show some preliminary evidence for a first-daughter effect, since the coefficient for the eldest-daughter variable is positive for both the ‘Parents’ and the ‘Fathers’ models, although it is has greater statistically significance exactly where we would expect first-daughter effects to occur, i.e. only among fathers.

**Fig 2:**
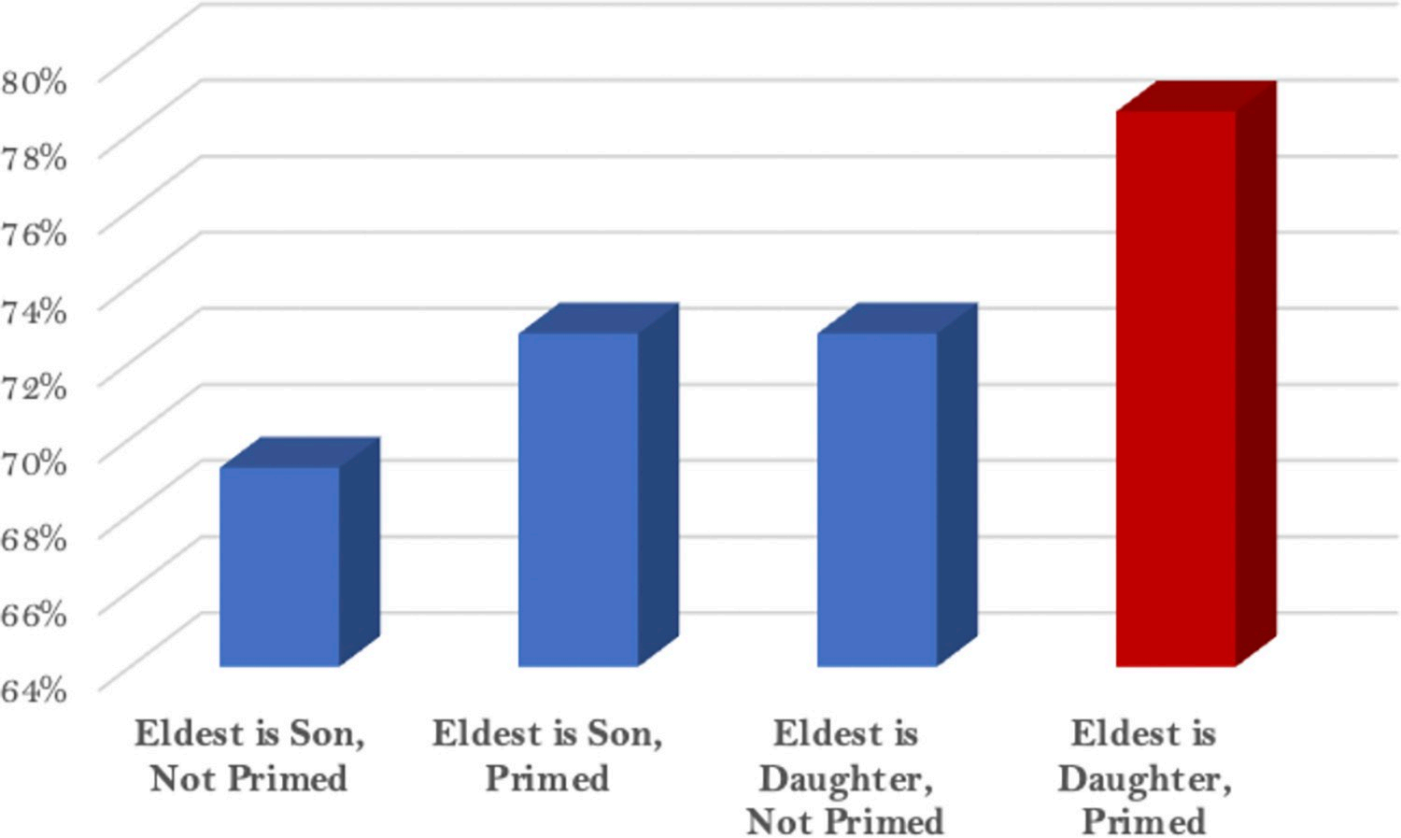
Comparative average responses among Afghan fathers to the question ‘I believe achieving human rights for women is among the top priorities for the future of my country’.

In an additional step, we focus on the respondent sample of parents only, so that the estimation of those with eldest-daughters are essentially a random assignment, due to the randomness of the sex ratio in the population. The reference case is respondents with an eldest child who is a son. In the ‘Parents’ model, we run the same logistic regression model as specified above, but restrict the sample to all parents. As Fig 2 illustrates via a coefficients plot, the eldest-daughter effect is positive but not statistically significant at conventional levels.

We then ran a range of other models which were restricted only to fathers but also included our ‘priming’ variable in some models, and not others. Specifically, in the ‘Primed Fathers’ model, we introduced the variable of the randomly assigned order of the “if you have children, what is their gender” question. This variable allows us to examine whether ‘priming’ respondents with this question made any difference to their surveyed attitudes. As illustrated in Table 1 below, in this ‘Primed Fathers’ model, we find a statistically significant association, which indicates support for the ‘priming’ mechanism compared to the ‘experiential’ mechanism.

**Table 1:** Logistic regression results estimating support for women’s rights.

	(1)	(2)	(3)	(4)
		Just Fathers		
	All Respondents	Fathers, Gender of Eldest Child	Fathers, Gender of Eldest Child, Primed	Fathers, Interactions
Male Respondent	-0.21*			
	(0.12)			
Support for Government	-0.29***	-0.24***	-0.25***	-0.25***
	(0.03)	(0.05)	(0.05)	(0.05)
Religiosity	-0.10*	-0.19**	-0.19**	-0.19**
	(0.05)	(0.09)	(0.09)	(0.09)
HR Violations a Top Priority	0.55***	0.54***	0.54***	0.54***
	(0.04)	(0.06)	(0.06)	(0.06)
Eldest is Daughter		0.24	0.23	
		(0.15)	(0.15)	
Primed			0.30**	
			(0.15)	
Eldest is Son, Primed				0.24
				(0.19)
Eldest is Daughter, not Primed				0.15
				(0.21)
Eldest is Daughter, Primed				0.54**
				(0.21)
Observations	2938	1136	1136	1136
Pseudo *R*^2^	0.119	0.114	0.117	0.118
*BIC*	3130.34	1249.87	1252.60	1259.36

*Note*: Standard errors in parentheses, * *p* < 0.10, ** *p* < 0.05, *** *p* < 0.01

Table 1 reports the estimated coefficients, with standard errors in parentheses, for four different models. The first model is the ‘All Respondents’ model visualized above, for the purposes of comparison. We omit the large range of control variables mentioned above, with the exception of government support, religiosity, and the prioritization of human rights violation question. More detailed regression tables are available in the S1 Appendix.

Model 1 shows what the results look like, in tabular form, for the ‘All respondents’ variable. All subsequent models restrict to fathers only. Model 2 then shows the positive but weakly significant results when we include a variable for the eldest child being a daughter. This suggests some qualified evidence in support of the ‘experiential’ mechanism, but the effect is not statistically significant at conventional levels.

A key step to identify which mechanism might be at work comes in Models 3 and 4. To investigate the ‘priming’ mechanism, in Model 3 we introduce a variable to indicate whether or not a respondent was ‘primed’ with the child gender question before they answered the focal question about women’s human rights (i.e., the key outcome of interest). The effect of priming is positive and is statistically significant–this indicates strong support for the ‘priming’ mechanism generating the first-daughter effect. Model 4 then introduces different categorical interactions, each representing a different combination of being primed on the one hand, and having a son or daughter, on the other (the baseline configuration are fathers who have sons and were not primed). For fathers whose eldest child is a son, there is no statistically significant effect. For fathers with an eldest daughter who were not primed, the effect is weaker and is also not statistically significant. However, when a father has an eldest daughter and was primed, the effect is positive and larger than other estimated effects, and the effect is also statistically significant. Thus Model 4 shows yet stronger and more focused support for the ‘priming’ mechanism driving the first daughter effect.

These findings show support for the eldest-daughter effect and also helpfully differentiate which of the key mechanisms may be at play. Specifically, our findings suggest that it is not simply *having* an eldest daughter that matters, but also the act of *thinking* about the gender of one’s eldest child in the context of considerations about gender equality in one’s society. In short, it is not the experiential mechanism alone that drives the eldest-daughter effect, but rather that mechanism in combination with the “priming” mechanism. As described in the S1 Appendix, we replicated the latter two models but for mothers only. ‘Just Mothers’ or ‘Primed Mothers’ have no statistically significant association.

We visualize these findings in Fig 2 below, which shows the combinations of Afghan fathers whether having an eldest daughter and / or being primed. The marginal effects from our regression results indicate that being primed when a father has an eldest son has no statistically significant effect on fathers in Taliban-controlled Afghanistan. Additionally, if a father simply *has* an eldest daughter, but was *not primed* by the child-gender question, the null result reported earlier remains. In other words, the mechanism of experience alone does not appear to drive the first-daughter effect. Fathers of eldest daughters need to be *primed* in order for the effect to really manifest. Relative to a father who was not primed and who has an eldest son, which our models estimate to have a mean estimate of 71%, a father who is primed about their eldest daughter has a mean estimate of 80%, which is a sizable difference.

We checked for covariate imbalance through coarsened exact matching [56, 57] and assessed whether our results were sensitive to covariate imbalance between the ‘treatment’ and ‘control’ groups, of our main effect. Specifically, we coarsened-exact matched on the variables that could plausibly differ across control and treatment groups specifically because of survey ‘dropout’ effects, whereby the ideological composition primed by the child-gender question gets skewed because of differential dropout rates across the primed and non-primed groups. We matched on support for the Taliban government, religiosity, levels of education and urban-rural. The effect persists. We also did a similar coarsened exact matching exercise for the non-attitudinal demographic questions, such as urban-rural, placement on the economic ladder, age (under/above 30), etc. The effect held at the same p-value range.

There is support for this effect not only in the statistical analysis of respondents’ answers to closed-ended questions, but in the qualitative analysis of their open-ended explanations for the responses they gave. Using skip sequencing, the answer to Question 9 on women’s human rights as a national priority led the respondent to one of three separate open-ended prompts [58]. Those answering, “strongly disagree” or “disagree” (20% of the total) received Question 10a, which read, “If you wish, name three things you believe are a higher priority than human rights for women and why.” Those answering “undecided” (35% of the total) received Question 10b, which read, “If you wish, tell us more about why you are undecided on this question.” Those answering “agree” or “strongly agree” (65% of the total) received Question 10c, which read, “In your own words, what would ‘achieving human rights for women’ look like to you?”.

Those “agreeing” (Q10C) who chose to describe their vision of women’s human rights answered the question in a number of ways. 35% of these respondents simply listed the women’s human rights that were most important to them, with the “Right to Work” and “Right to Education” being the most frequent responses. Less frequent themes in the data included the expressed idea that women’s rights would strengthen society (10%), that women’s rights were not possible under the Taliban (11%), that international intervention was required to protect women’s rights (5%), or that women’s rights should take an Islamic form (16%). Among this group, when we compare fathers of eldest daughters who were “primed” to the general pool of respondents who “agreed” with women’s human rights, we see that “primed” fathers are 4 percentage points likelier to mention “equality” as a right of its own in their open-ended remarks. They are also 2% more likely to say that women’s rights strengthen society, and 3% more likely to call for international pressure to persuade the Taliban to allow women and girls their rights. Some examples of these ideas in fathers’ own words include:

“Women fulfill human rights and deprivation of human rights is a form of disobedience to my Lord. I will be proud of myself that I will fight for women’s rights.”

“Afghan women should have the right to work, education and other legal issues because they are who we are. Women’s rights can not be ignored in any way! And I ask you please! Do something, do not let it continue like this!”

“Women make up half of Afghanistan’s society… let them try to work like Afghan men alongside men for the development and prosperity of this country. Which in the present age is an urgent need of us Afghans.”

Among those who responded to Q9 by saying they were “Undecided,” general themes in respondent explanations of their answers included “Other Issues More Important” including food, security, or the rights of all Afghans: one respondents wrote they were undecided on the women’s rights question “Because it is not only the rights of women that are at stake, the rights of all Afghan citizens must be respected;” another wrote “Afghanistan’s economy is at a standstill. There are atrocities. There are no jobs. People are starving. Children and young people are missing.”) Secondly, many citizens in this category described genuine uncertainty or fear in even answering the question: a large number stated some variant of “I just don’t know” (for example: “I remained indecisive about this question because I can’t answer this question in the current situation” or “Because I do not know what you mean by human rights?”); others stated “I’m Afraid To Answer” (for example, “I’m afraid my family’s life will be in danger”; and “I’m worried, I can’t say.”). Another 23% of the “undecided” respondents described feeling sympathetic toward women’s human rights but “resigned” that very little could be done about it under the Taliban (as examples: “The current situation in Afghanistan does not allow women to demand their rights” or “Because the government does not want to give freedom like the rest of the world” or “There is a gap between the Taliban and the aspirants for democracy”). 25% of “undecided” respondents mentioned Islam as a reason for their ambivalence (for example: “Because Islam has stated its rights for women in the Qu’ran” or “I am indecisive because I do not want women to be deprived of education, nor do I want them to be like the previous system, but to be equal to Sharia and within the framework of Sharia.”) However, among primed fathers as a specific group, these percentages changed: a full 46% of primed fathers of eldest daughters expressed feeling “Sympathetic But Resigned” versus the baseline 23%; and only 13% of primed fathers mentioned "Islam” as a source of their ambivalence on women’s rights versus a baseline 25%.

Even among those who selected the “disagree” or “strongly disagree” options when asked about women’s human rights as a national priority, priming fathers of eldest daughters as a group to actually think about their daughters explains the reasons for this answer in distinct ways. When asked to describe what they felt was more important than women’s rights, the percentage of primed fathers choosing “Democracy/Freedom” as the most important priority rose to 21% compared to 15% among the general population in this category; 32% of primed fathers mentioned “Economy/Jobs” versus only 19% of the general population in this category; and 10% of primed fathers mentioned “Security/Safety” versus 6% of the wider respondent pool. By contrast, references to Islam and Religion as a Priority were lower among primed fathers, as a rationale for disagreeing on women’s human rights, compared to the wider pool of respondents in this category.

Thus, across all categories of respondents regardless of how they ‘scored’ on attitudes toward women’s rights as a national priority, we see a marked difference for fathers of eldest daughters, particularly those who were primed to think about the gender of their eldest child before answering the other survey questions. In short, when Afghan fathers are primed about their eldest child being a daughter, their subjectivity changes.

Based on this evidence, we can conclude that there is a range of empirical evidence for an eldest-daughter effect in Afghanistan, and in ways actually conducive to human rights advocacy. The very fact of having an eldest daughter–what we call the ‘experiential mechanism’ is not by itself robustly associated with support for the human rights of women. We do find evidence in support of a ‘priming mechanism’, which entails not only the experience of having an eldest daughter, but actively recalling it when considering other kinds of questions. It is only when Afghan men with daughters are ‘primed’ with the child-gender question that their support for the human rights of women systematically increases. As we discuss below, this particular ‘priming mechanism’ is hugely significant because it is actually something that those interested in changing attitudes and policies in Afghanistan can affect, whereas the actual sex ratios of a population are not subject to manipulation.

## Discussion

Our study of attitudes toward women’s equality in Afghanistan shows strong public support for the human rights of women and girls, even among those Afghans who say they support the Taliban. Unsurprisingly, controlling for other variables, positive attitudes toward women’s rights are predicted by being a woman, and by living in urban areas; negative attitudes toward women’s equality are predicted by support for the current government, religiosity, and lower socio-economic status. Controlling for these variables, however, we also found that Afghan fathers with eldest daughters *who were asked to answer a question about the gender of their eldest child* ***before*** *answering the question about women’s rights* had a much higher likelihood to report feeling strongly in favor of women’s rights as a top priority for the future of the nation. Even those who did not gave explanations of their answers more indicative of a concern for human rights in general, and women’s human rights in particular, than other Afghan men. This finding is conversant with the idea that attitudes toward gender equality can be manipulable, even among unlikely populations [59].

There are limitations to our study that we should note. First, our findings are limited to the internet-using population. However, our use of RIWI’s novel online data collection technology allowed us to reach the most diverse Web-population possible using nonprobability sampling, serving as a strength relative to other online data collection platforms (e.g., panel-based research). All Web-users in Afghanistan had a chance of encountering this survey, thus minimizing self-selection biases, and maximizing our reach to a broader set of respondents who might otherwise not take part in research. While our data and analysis are obviously restricted to the subset of the Afghan population who uses the internet, this might also be the very population that human rights organizations might be able to reach. Confidence in our main finding is also informed by the fact that both the assignment of a child’s gender and the priming experiment were both randomly assigned.

Second, we observed a high drop-off in respondents from opt-in to survey completion. While we cannot know the exact drivers of the variation in survey completion, we do know that, because of the possibility to skip certain questions, basic demographic information, such as the gender of the respondent, was much more complete compared to other questions related to attitudes. For example, almost 26,000 respondents provided information on their gender and age, while 7513 completed the question regarding the human rights of women. While it is highly likely that this divergence was due to the political sensitivity of some questions versus others, we note that the number of responses on the human rights of women question was higher than other potentially politically sensitive questions (e.g., importance of religion = 2933; placement on the ‘economic ladder’ = 2989). Meanwhile, the question support for the Taliban government itself had an even higher completion rate, at 10,471.

Our findings, nonetheless, are relevant both theoretically and for the practitioner community. Despite great concern for improving the well-being of women and girls, the scientific literature on gender and conflict has said little about how to target which specific men as allies for women in violently divided societies recovering from armed conflict or living under gender authoritarian regimes. At the same time, the literature on determinants of male attitudes toward gender equality involves few studies in these contexts. Within the literature on the first father effect, for example, ours is only the fourth to analyze a non-Western country context (see [23] re. Turkey, [22] re. South Africa and [21] re. Japan) and the first ever to disaggregate the priming effect of being asked to think about one’s daughter (in the context of a survey about the future of the country) from the social effect of actually having an eldest daughter. Our findings lend qualified support to the idea that fathers of eldest daughters may have, or are susceptible to having triggered, more liberal attitudes toward gender equality.

In the context of Taliban-controlled Afghanistan, this finding has practical implications as well. Human rights organizations can find ways, even subtle ones, to appeal to fathers of eldest daughters (or perhaps fathers of daughters per se, knowing this will reach these fathers as well) as a demographic. Humanitarian organizations could, for example, include images of Afghan fathers and daughters in promotional materials, particularly online or social media initiatives, which would reach the population on whom this study is based. Local civil society organizations concerned with women’s rights could consider targeting fathers with eldest daughters for alliances or assistance with programming initiatives designed to support women and girls. Or they could be trained to bear in mind the idea that the individual in any given household who might be the best ally for a given women’s rights initiative could well be the father when the eldest child is a girl.

## Supporting information

S1 Appendix Appendix for ‘The first daughter’ effect: Human rights advocacy and attitudes toward gender equality in Taliban-controlled Afghanistan. (DOCX)

S1 File Inclusivity in global research. (DOCX)
