# Supplementary material for: The “First daughter” effect: Human rights advocacy and attitudes toward gender equality in Taliban-controlled Afghanistan
Source: PLoS One. 2024 Jul 17;19(7):e0298812. doi: 10.1371/journal.pone.0298812 (PMC11253973; doi:10.1371/journal.pone.0298812)
Supplement: S1 Appendix — (DOCX) [file pone.0298812.s001.docx]

Appendix for ‘The First Daughter’ Effect: Human Rights Advocacy and Attitudes Toward Gender Equality in Taliban-Controlled Afghanistan

Table A1:

Full Regression Table Corresponding to Models in Figure 1

This table corresponds to each of the Models visualized in Figure 1 of the manuscript. We excluded from that visualization a number of dummy variables which we include here. Each regression model includes survey weights.

|  | (1) | (2) | (3) |
| --- | --- | --- | --- |
|  | All Respondents | Parents | Fathers |
|  |  |  |  |
| Support for Govt | -0.28^***^ | -0.22^***^ | -0.26^***^ |
|  | (0.04) | (0.05) | (0.05) |
| Religiosity | -0.09 | -0.20^**^ | -0.21^**^ |
|  | (0.06) | (0.08) | (0.09) |
| HR Violations a Top Priority | 0.54^***^ | 0.54^***^ | 0.50^***^ |
|  | (0.04) | (0.06) | (0.06) |
| Lives in Urban Area | 0.31^**^ | 0.18 | 0.06 |
|  | (0.13) | (0.19) | (0.20) |
| Economic Ladder | -0.06^***^ | -0.03 | -0.04 |
|  | (0.02) | (0.03) | (0.03) |
| Age Over 30 | -0.11 | -0.14 | 0.21 |
|  | (0.13) | (0.15) | (0.16) |
| University | -0.02 | -0.13 | -0.21 |
|  | (0.11) | (0.16) | (0.16) |
| Single | -0.46^***^ | -1.26^***^ | -0.70^**^ |
|  | (0.16) | (0.25) | (0.28) |
| Widowed, Divorced, or Separated | -0.90^***^ | -0.84^**^ | -0.87^*^ |
|  | (0.31) | (0.42) | (0.50) |
| Wave | -0.10 | -0.10 | -0.11 |
|  | (0.06) | (0.10) | (0.10) |
| Has Child | -0.29^*^ |  |  |
|  | (0.15) |  |  |
| Male Respondent | -0.14 | 0.06 |  |
|  | (0.13) | (0.20) |  |
| Eldest is Daughter |  | 0.21 | 0.26^*^ |
|  |  | (0.15) | (0.16) |
| Observations | 2938 | 1344 | 1136 |
| Pseudo *R*^2^ | 0.123 | 0.128 | 0.115 |
| *BIC* | 2679.01 | 1461.61 | 1065.64 |

Notes: Standard errors in parentheses, ^*^ *p* < 0.10, ^**^ *p* < 0.05, ^***^ *p* < 0.01

**Figures A2 and Table A2**

We include in the Appendix additional regressions models which restrict the sample to only Afghan parents, just fathers, with and without priming, as well as just mothers, with and without priming. As expected by the ‘priming’ mechanism, we find that only fathers who were primed to have a statistically significant and positive association with the support for human rights of women.

Figure A2:

Coefficients Plot of Focal Variables ‘Eldest is Daughter’ and ‘Priming’ question, across a range of models

Figure A2 visualizes the estimated effect of the two focal variables, the eldest daughter variable and the priming variable. Table A2 below shows the full regression results for each variable used in these models.

|  | *(1)*  *All Respondents* | *(2)*  *Only Fathers* | *(3)*  *Primed Fathers* | *(4)*  *Only Mothers* | *(5)*  *Primed Mothers* |
| --- | --- | --- | --- | --- | --- |
|  |  |  |  |  |  |
| Respondent is a Woman | 0.04 |  |  |  |  |
|  | (0.19) |  |  |  |  |
| Eldest is Daughter | 0.18 | 0.23 | 0.21 | -0.05 | -0.03 |
|  | (0.14) | (0.15) | (0.15) | (0.37) | (0.37) |
| Support for Govt | -0.21^***^ | -0.25^***^ | -0.25^***^ | -0.05 | -0.06 |
|  | (0.04) | (0.05) | (0.05) | (0.13) | (0.12) |
| Religiosity | -0.17^**^ | -0.18^**^ | -0.17^*^ | -0.02 | -0.04 |
|  | (0.08) | (0.09) | (0.09) | (0.18) | (0.18) |
| HR Violations a Top Priority | 0.54^***^ | 0.53^***^ | 0.53^***^ | 0.61^***^ | 0.60^***^ |
|  | (0.05) | (0.06) | (0.06) | (0.14) | (0.14) |
| Lives in Urban Area | -0.00 | -0.04 | -0.04 | 0.14 | 0.13 |
|  | (0.17) | (0.19) | (0.19) | (0.40) | (0.40) |
| Economic Ladder | -0.03 | -0.03 | -0.03 | -0.02 | -0.02 |
|  | (0.03) | (0.03) | (0.03) | (0.08) | (0.07) |
| Aged 25-34 | 0.19 | 0.30 | 0.31 | -0.02 | -0.02 |
|  | (0.20) | (0.22) | (0.22) | (0.59) | (0.59) |
| Aged 35-44 | 0.12 | 0.37 | 0.36 | -0.92 | -0.88 |
|  | (0.22) | (0.25) | (0.25) | (0.56) | (0.56) |
| Aged 45-54 | 0.53^*^ | 1.00^***^ | 0.98^***^ | -1.38^*^ | -1.33^*^ |
|  | (0.32) | (0.38) | (0.37) | (0.75) | (0.74) |
| Aged 55-64 | 0.67 | 0.60 | 0.64 | 0.93 | 0.93 |
|  | (0.48) | (0.53) | (0.53) | (1.00) | (0.96) |
| Aged 65+ | 0.15 | 0.40 | 0.40 | -0.64 | -0.56 |
|  | (0.39) | (0.44) | (0.44) | (0.82) | (0.81) |
| Govt Elementary | 0.08 | 0.24 | 0.24 | -0.70^*^ | -0.71^*^ |
|  | (0.18) | (0.21) | (0.21) | (0.42) | (0.43) |
| Private Elementary | -0.38 | -0.14 | -0.15 | -1.19 | -1.17 |
|  | (0.39) | (0.46) | (0.47) | (0.87) | (0.84) |
| Technical | 0.75 | 0.50 | 0.57 | 1.47 | 1.52 |
|  | (0.46) | (0.46) | (0.47) | (1.61) | (1.66) |
| Govt High | 0.19 | 0.23 | 0.21 | -0.17 | -0.21 |
|  | (0.19) | (0.20) | (0.20) | (0.58) | (0.58) |
| Private High | 0.36 | 0.32 | 0.29 | 0.89 | 0.82 |
|  | (0.40) | (0.46) | (0.46) | (1.02) | (0.98) |
| Single | -0.73^***^ | -0.52^*^ | -0.58^**^ | -1.52^***^ | -1.47^***^ |
|  | (0.24) | (0.28) | (0.28) | (0.50) | (0.50) |
| Widowed, Divorced, or Separated | -1.01^***^ | -0.81^*^ | -0.85^*^ | -1.10 | -1.08 |
|  | (0.36) | (0.44) | (0.45) | (0.69) | (0.71) |
| Wave of Survey | -0.02 | -0.02 | -0.02 | 0.09 | 0.10 |
|  | (0.08) | (0.09) | (0.09) | (0.23) | (0.23) |
| Primed by Child-Gender Question |  |  | 0.31^**^ |  | -0.35 |
|  |  |  | (0.15) |  | (0.36) |
| Observations | 1344 | 1136 | 1136 | 208 | 208 |
| Pseudo *R*^2^ | 0.119 | 0.120 | 0.123 | 0.211 | 0.214 |
| *BIC* | 1532.67 | 1298.65 | 1301.26 | 305.36 | 309.81 |

Notes: Standard errors in parentheses, ^*^ *p* < 0.10, ^**^ *p* < 0.05, ^***^ *p* < 0.01
